# HCC with tumor thrombus entering the right atrium and inferior vena cava treated by percutaneous ablation

**DOI:** 10.1186/s12893-017-0217-y

**Published:** 2017-02-28

**Authors:** Wei Li, Yang Wang, Wenfeng Gao, Jiasheng Zheng

**Affiliations:** Center of Interventional Oncology and Liver Diseases, Beijing You’an Hospital, Capital Medical University, 8 Xitoutiao, Youanmenwai St., Fengtai Dist., Beijing, 100069 People’s Republic of China

**Keywords:** Hepatocellular carcinoma, Percutaneous, Ablation, Tumor thrombus, Right atrium, Case report

## Abstract

**Background:**

In the advanced stages of hepatocellular carcinoma (HCC), a tumor thrombus (TT) can form in the portal or hepatic vein. The management of patients with advanced HCC and a TT extending into the right atrium (RA) and inferior vena cava (IVC) is extremely difficult and risky.

**Case presentation:**

We report the case of a patient with HCC and a large TT (85 × 45 mm) extending into the RA through the hepatic vein and IVC, which is very rare. We performed percutaneous microwave ablation of the TT and the two intrahepatic tumors (maximum diameter, 57 mm). The treatment shrank the tumors, and the patient is in good condition and has survived for 16 months thus far. A literature review was also performed. This is the first such case to be treated with percutaneous microwave ablation.

**Conclusion:**

The outcomes in this case suggest that percutaneous ablation is useful for the treatment of TT extending into the RA and IVC in patients with HCC.

## Background

Hepatocellular carcinoma (HCC) is one of the most common malignant tumors and the second leading cause of cancer-related deaths [1]. HCC is a highly progressive cancer with a high rate of metastasis. Furthermore, tumor thrombus (TT) formation in the portal or hepatic vein is common in the advanced stages of HCC [2]. When the tumor thrombus invades the inferior vena cava (IVC) and right atrium (RA), the prognosis is usually very poor, since the condition may lead to cardiac failure or pulmonary embolization [3–5]. Furthermore, the treatment options at this stage are limited and not very effective. The general treatment of choice is major surgery with cardiopulmonary bypass, which is dangerous, risky, and expensive. In addition, many patients at this stage of HCC cannot tolerate such an operation.

We report the case of a patient with HCC associated with a tumor thrombus extending into the RA that was treated with percutaneous microwave ablation (MWA). To our knowledge, this is the first reported case in which minimally invasive percutaneous ablation was used to treat an HCC patient with a tumor thrombus in the RA.

## Case presentation

A 73-year-old man, who had been hepatitis B virus (HBV) positive for around 40 years, was diagnosed with primary HCC and cirrhosis. He was asymptomatic at the time, and the tumor was detected during a routine examination. He was admitted to our hospital, where a physical examination revealed no abnormality. He had mild hypertension, which was well controlled. He had received no treatment for HCC before admission to our hospital.

**Fig. 1:**
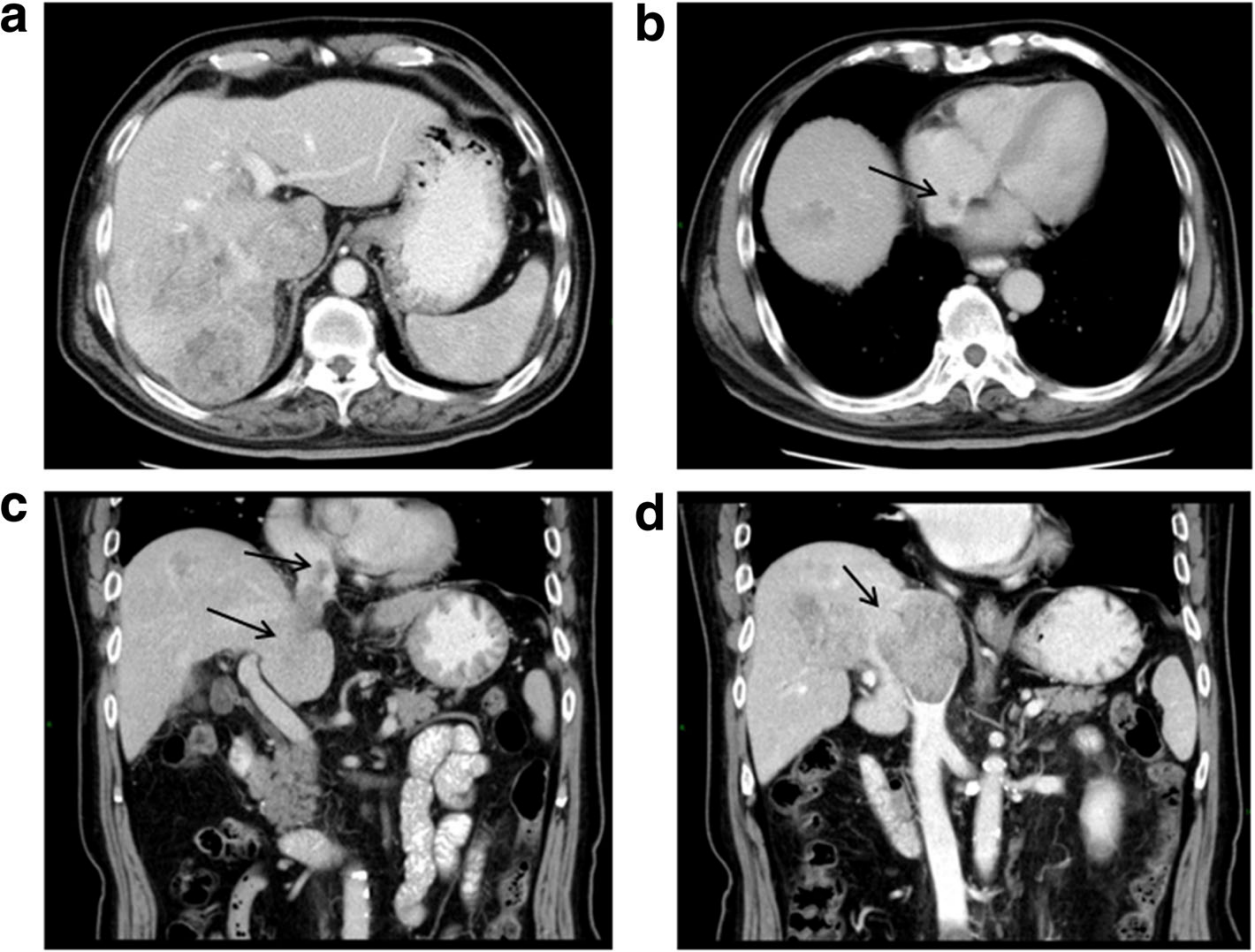
Pre-operative CT scanning. **a** Enhanced CT of the abdominal region showed multiple tumor nodules at the right lobe of the liver and tumor thrombus in IVC. **b** Enhanced CT showed an accompanying tumor thrombus in RA (*black arrow*). **c, d** Enhanced CT showed tumor thrombus in IVC and RA (*black arrow*). CT: computed tomography, RA: right atrium, IVC: inferior vena cava

**Fig. 2:**
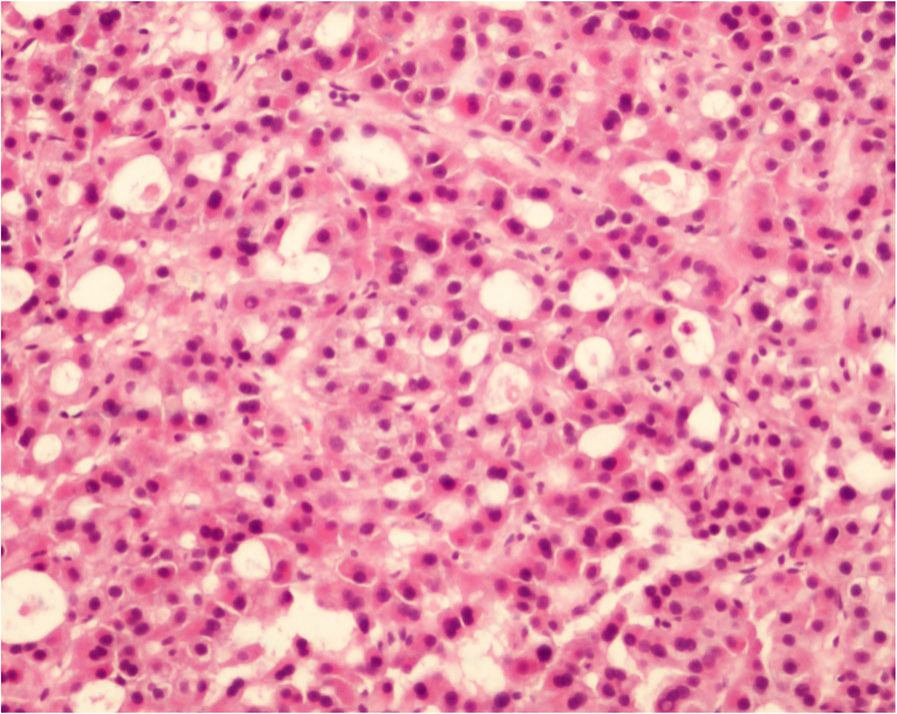
Pathology of the thrombus. Hematoxylin-eosin staining of the tumor thrombus revealed tumor cells consistent with HCC of the patient (200X). HCC: hepatocellular carcinoma

Pre-operative computed tomography (CT) revealed multiple tumor nodules in segments VII and VIII of the liver (Fig. 1a), with an accompanying tumor thrombus growing from the accessory hepatic vein, and through the IVC, and into the RA (Fig. 1b, c, and d). The maximal diameters of the tumor nodules and thrombus were 57 and 45 mm, respectively. The length of the thrombus was 85 mm. Pre-operative laboratory tests revealed the following: alpha-fetoprotein (AFP), 266.6 ng/mL; AFP-L3, 22.7 ng/mL; HBV-DNA, 4.58 × 10^6^ copies/mL; Eastern Cooperative Oncology Group score, 0; and Child-Pugh grade, A. His body–mass index was 23.88. Pre-operative histological examination of the tumor confirmed HCC (Fig. 2).

Patients with RA tumor thrombus usually have a poor prognosis. Sudden death can occur due to right heart failure or pulmonary embolization. However, our patient refused surgical treatment. We therefore offered him the option of percutaneous MWA to which he consented.

**Fig. 3:**
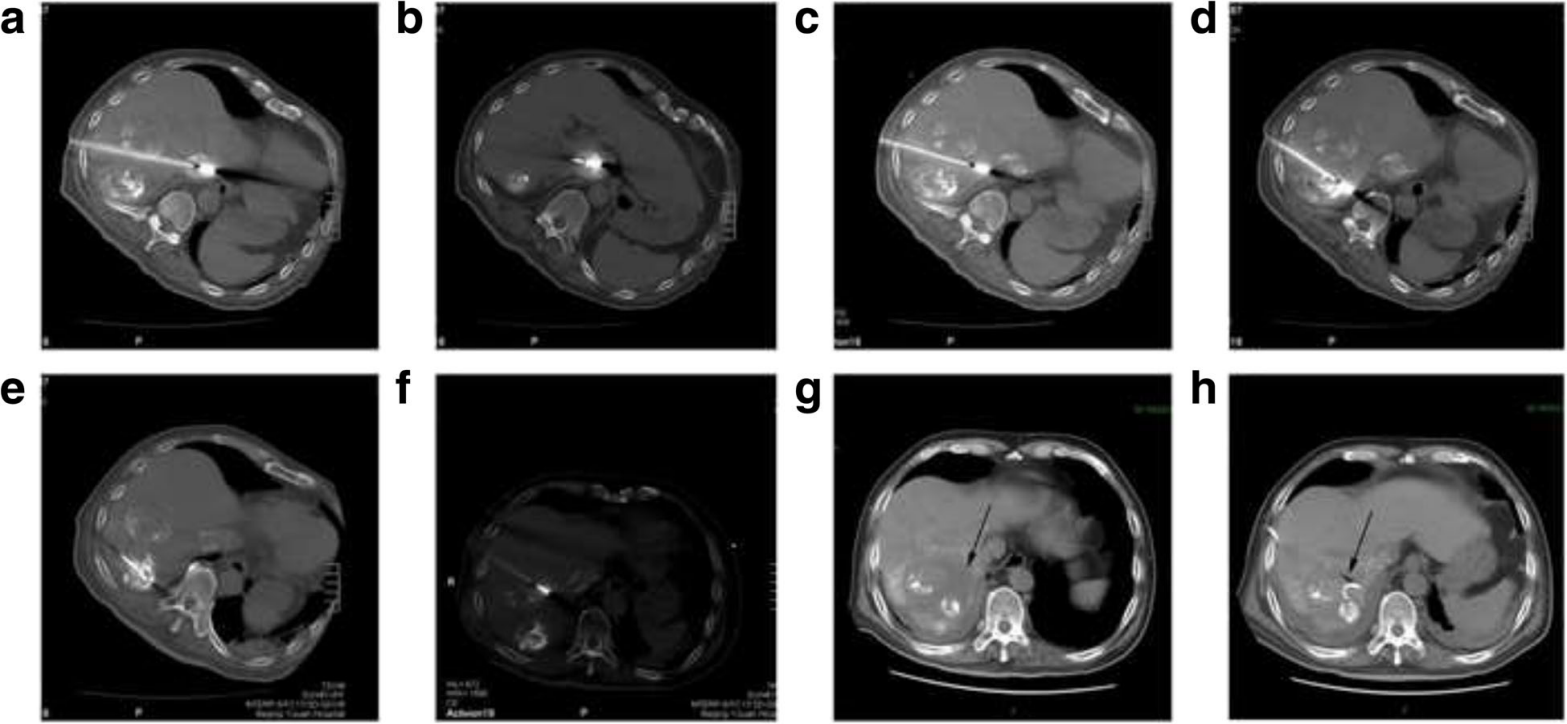
Intra-operative CT scanning and post-operative biloma. **a** CT scanning showed ablation of the upper part of tumor thrombus in IVC and RA. **b** CT scanning showed ablation of the lower part of tumor thrombus in IVC and RA. **c** CT scanning showed ablation of the basal part of tumor thrombus in the hepatic vein. **d** CT scanning showed ablation of the distal part of tumor thrombus in the hepatic vein. **d** CT scanning showed ablation of the distal part of tumor thrombus in the hepatic vein. **e** CT scanning showed ablation of the tumor in segment VII. **f** CT scanning showed ablation of the tumor in segment VIII. **g** CT scanning showed biloma which was a fluid collection in the space surrounding the necrotic tissue of the ablation zone at segment VIII (*black arrow*). **h** CT scanning showed significant shrinkage of biloma 10 days after drainage (*black arrow*). CT: computed tomography, RA: right atrium, IVC: inferior vena cava

Pre-operative transcatheter arterial chemoembolization (TACE) was performed to label the tumor and thrombus, so that they could be easily targeted on CT scans without contrast enhancement. MWA was performed under local anesthesia with 1% lidocaine. The best puncture path to reach the TT is through the liver parenchyma, without involving the tumor tissue and important vessels. Therefore, the left-lateral position was selected to expose the intersection of the ninth intercostal space and posterior axillary line, which we considered to be the best puncture point. A 22-G puncture needle was used to lead the microwave electrode to the target. The angle and depth of each puncture was calculated using intraoperative CT scans to ensure that the needle was in the correct position and direction. After performing CT and calculating and adjusting the puncture angle four times, the microwave electrode was successfully advanced up to the targeted upper part of the TT in the IVC (Fig. 3a) without any intra-operative complications. A microwave generator (Vison Medical, Nanjing, P.R. China), which provided a frequency of 2450 MHz and a power output of 60 W, was used to ablate the upper part of the TT for 6 min. Then, the needle was withdrawn 7 cm until it was back in the liver parenchyma. Sufficient space was left for the adjustment of the needle heading the lower part of the TT in the IVC. The lower part of the thrombus was then ablated using a power output of 70 W for 6 min (Fig. 3b). Afterwards, the basal part (Fig. 3c) and distal part (Fig. 3d) of the tumor thrombus in the accessory hepatic vein and the tumor in segment VII (Fig. 3e) were ablated sequentially. The ablation procedure was repeated twice to ablate the tumor in segment VIII (Fig. 3f) and achieve satisfactory necrosis. The second and third ablations were performed 8 days apart within 21 days of the first procedure, with the exact timing depending on the recovery of liver function. No complications apart from mild-to-intermediate liver dysfunction were observed, and the patient was discharged 5 days after the last ablation procedure.

**Fig. 4:**
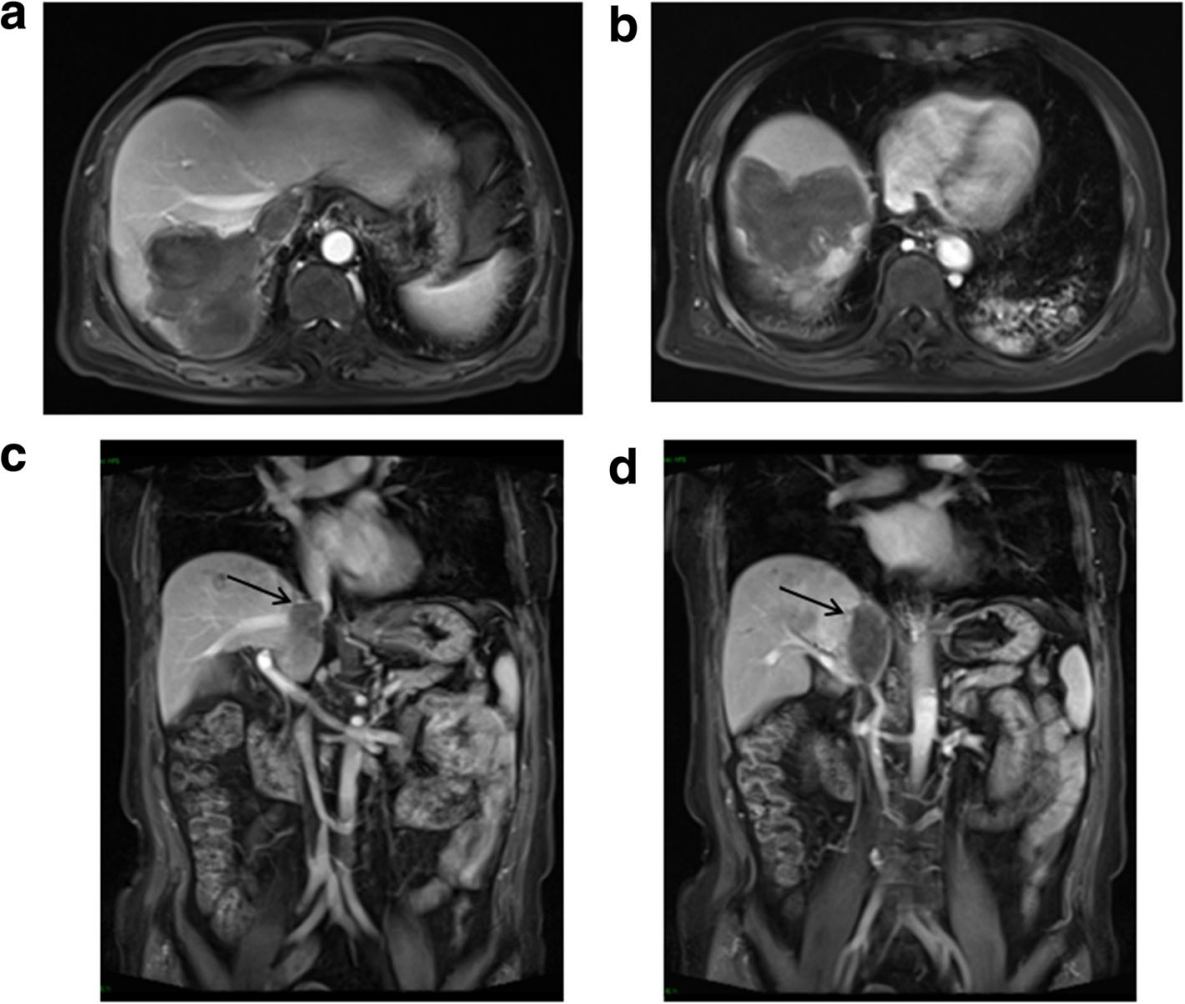
MRI at 1 month post-operatively. **a**, **b** Enhanced MRI of the abdominal region showed ablated tumor area at segment VII and VIII. **c**, **d** Coronal sequence of enhanced MRI of the abdominal region showed the shrinkage of the tumor thrombus in IVC and RA (*black arrow*). MRI: magnetic resonance imaging, RA: right atrium, IVC: inferior vena cava

Three months later, an intrahepatic tumor recurrence was detected. TACE and salvage MWA were repeated, and the tumors were completely ablated. The patient developed fever and chills on days 7 after this treatment, and CT revealed a biloma in segment VIII (Fig. 3g). A drainage tube was inserted. Within 10 days, the fever was resolved, and the biloma showed significant shrinkage on CT (Fig. 3h). The patient complained of no discomfort afterwards and was discharged one month after the salvage ablation. At the time of discharge, his AFP was 2.66 ng/mL, and magnetic resonance imaging (MRI) showed necrosis of the tumors in segment VII (Fig. 4a) and VIII (Fig. 4b) and shrinkage of the tumor thrombus in the RA (Fig. 4c) and IVC (Fig. 4d).

**Fig. 5:**
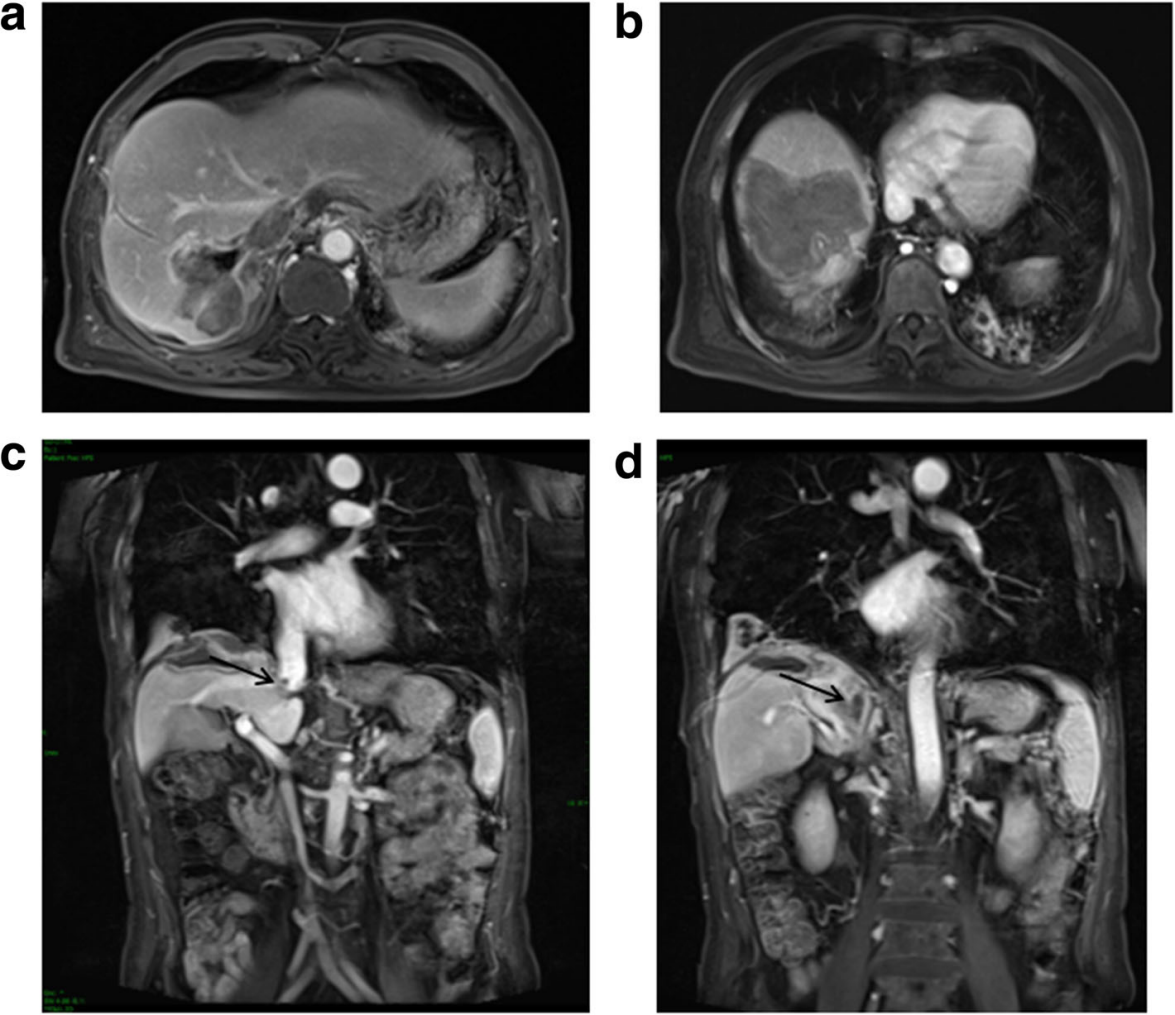
MRI at 5 month post-operatively. **a**, **b** Enhanced MRI of the abdominal region showed further shrunk ablated tumor area at segment VII and VIII. **c**, **d** Coronal sequence of enhanced MRI of the abdominal region showed the further shrinkage of the tumor thrombus (*black arrow*). MRI: magnetic resonance imaging, RA: right atrium, IVC: inferior vena cava

Enhanced MRI was done during routine follow-up. With proliferation of the hepatic parenchyma and absorption of the necrotic tissue, further shrinkage of the ablated tumors in segment VII (Fig. 5a) and VIII (Fig. 5b) as well as the tumor thrombus in RA (Fig. 5c) and IVC (Fig. 5d) was seen 5 months after treatment. The patient has survived and been followed up for 16 months since the diagnosis of RA thrombus. Currently, he is alive without any further recurrence.

## Discussion

Only 0.67–4.1% of HCC patients develop a tumor thrombus extending into the RA [6, 7]. In our patient, the accessory hepatic vein was invaded by the tumor all the way up to the RA. This is a very rare condition that carries a dismal prognosis, as it is associated with a high risk of systemic metastasis, acute pulmonary embolism, and heart failure [8–10].

The management of patients with advanced HCC and a tumor thrombus extending into the RA is difficult and risky. The prognosis is dismal if only supportive care is provided (median survival, 5 months) [11]. Surgical extraction of the thrombus and resection of the tumor appears to be the only effective treatment option. However, this is a major open surgery that commonly requires cardiopulmonary bypass [11–16]. Moreover, the operation involves incising the subcostal arch, RA, IVC, and all the way down to the hepatic venous root site [4]. Our patient refused surgery and preferred to undergo minimally invasive treatment. In the past, surgeons could extract only the tumor thrombus, owing to technical limitations and the post-operative survival was a mere 1–9 months (mean, 6 months) [4, 17]. With improvements in surgical techniques, it is now possible to simultaneously resect both the intrahepatic tumor and the RA tumor thrombus [18, 19]. The mean survival of patients after this operation, which involves cardiopulmonary bypass, has been reported to be 20 months (range, 18 days to 56 months) [14]. These results indicate the benefits of surgical treatment. However, patients with advanced HCC complicated with tumor thrombus in the IVC and RA are usually elderly and may not tolerate major open surgery to extract the thrombus. More over, there is a high risk of operative failure and complications related to general anesthesia in these patients. The high expense of major operations is also a factor.

TACE does not improve survival in HCC patients with tumor thrombus [11]. Here, we used TACE to label the tumor margins and help precisely ablate the thrombus and tumor masses. TACE and ablation therapy are minimally invasive; they do not require systemic anesthesia and can be tolerated by almost all HCC patients. Moreover, ablation therapy can be repeated to treat large tumors, multiple nodules, and tumor recurrences. This therapy has become increasingly important in the management of HCC, and is the treatment of choice in many other conditions [20]. Therefore, we decided to attempt ablative therapy in our patient. Since MWA is known for its larger ablation volumes, shorter duration, and resistance to the heat-sink effect as compared with radiofrequency ablation, we considered that using MWA would lessen the number of puncture procedures and thus reduce the risk of bleeding and ablation failure.

The intra-operative complications in our patient were mild and transient. No vascular thrombosis related with the ablation was observed. Abundant blood flow was present to dissipate the heat generated during ablation, and thus, the IVC was protected from being damaged by thermal ablation. The post-operative biloma and bile track infection in our patient were controlled within 10 days by using drainage and medical care. The patient has thus far survived for 16 months. He is in a good condition and is being routinely followed up.

A potential severe complication of our treatment is pulmonary embolism due to dislodgement of the ablated thrombus. However, this complication was not observed in our patient and has not yet been reported in the literature.

To our knowledge, this is the first report of the use of percutaneous ablation therapy for the management of an HCC patient with a tumor thrombus in the IVC and RA. The results in this patient indicate that this treatment may have comparable efficacy to conventional open surgery with less local trauma and without the need for general anesthesia.

## Conclusions

Percutaneous ablation therapy might represent a useful and promising therapeutic modality for HCC patients with tumor extension into the RA and IVC, including patients with advanced tumors and older patients. Large-scale clinical trials of HCC patients with RA/IVC tumor thrombosis treated with percutaneous ablation are ongoing in China.

## Abbreviations

AFP: Alpha-fetoprotein
BMI: Body mass index
CT: Computed tomography
HBV: Hepatitis B virus
HCC: Hepatocellular carcinoma
IVC: Inferior vena cava
RA: Right atrium
TACE: Transcatheter arterial chemoembolization
TT: Tumor thrombus
